# Post-dynamic Resistance Exercise Hypotension: Exploring Individual Responses and Predictors

**DOI:** 10.3389/fphys.2021.787444

**Published:** 2021-12-02

**Authors:** Rafael Y. Fecchio, Andreia C. C. Queiroz, Raphael Ritti-Dias, Eduardo Caldas Costa, Cláudia L. M. Forjaz

**Affiliations:** ^1^Exercise Hemodynamic Laboratory, School of Physical Education and Sport, University of São Paulo, São Paulo, Brazil; ^2^Physical Education Department, Federal University of Juiz de Fora, Governador Valadares, Brazil; ^3^Post-Graduate Program in Rehabilitation Science, University Nove de Julho, São Paulo, Brazil; ^4^Department of Physical Education, Federal University of Rio Grande do Norte, Natal, Brazil

**Keywords:** blood pressure, interindividual, between-subjects variation, moderators, strength exercise

## Abstract

**Background:** Post-dynamic resistance exercise hypotension (PREH) has been largely demonstrated. However, little is known regarding the interindividual variation of PREH magnitude and its predictors (i.e. factors of influence).

**Aims:** To assess the interindividual variation of PREH and its predictors related to the characteristics of the individuals and the exercise protocol.

**Methods**: This study retrospectively analysed data from 131 subjects included in seven controlled trials about PREH (including at least one dynamic resistance exercise and one control session) conducted by two research laboratories. The interindividual variation was assessed by the standard deviation of the individual responses (*SD*_IR_), and linear regression analyses were conducted to explore the predictors.

**Results:** PREH showed moderate interindividual variation for systolic (SBP, *SD*_IR_=4.4mmHg; 0.35 standardised units) and diastolic blood pressures (DBP, *SD*_IR_=3.6mmHg; 0.32 standardised units). For systolic PREH, multivariate regression analysis (*R*^2^=0.069) revealed higher baseline SBP (*B*=−0.157, *p*=0.008) and higher number of sets (*B*=−3.910, *p*=0.041) as significant predictors. For diastolic PREH, multivariate regression analysis (*R*^2^=0.174) revealed higher baseline DBP (*B*=−0.191, *p*=0.001) and higher exercise volume (i.e. number of exercises ^*^sets per exercise ^*^repetitions per sets >150; *B*=−4.212, *p*=0.001) as significant predictors.

**Conclusion:** PREH has a considerable interindividual variation. Greater PREH magnitude is observed in individuals with higher baseline blood pressure and after exercise protocols that comprehend higher number of sets and exercise volume.

## Introduction

Arterial blood pressure (BP) decreases significantly after the execution of different types of exercise (Brito et al., 2018), which has been called post-exercise hypotension (Kenney and Seals, 1993). A recent meta-analysis reported reductions of significant magnitude in clinic as well as 24-h ambulatory BPs after the execution of a single session of dynamic resistance exercise (Casonatto et al., 2016), showing that post-resistance exercise hypotension (PREH) may be clinically relevant. In addition, PREH magnitude seems to be associated with the chronic reductions observed in BP after a period of dynamic resistance training (Tibana et al., 2014; Moreira et al., 2016), suggesting that this acute response may be used to predict BP responsiveness to training.

However, PREH magnitude varies considerably among the studies in the literature. The meta-analysis conducted by Casonatto et al. (2016) reported a significant heterogeneity between the studies’ results for systolic and diastolic PREH. Some studies suggest that variation in PREH magnitude may be influenced by factors related to the population and/or the exercise protocol characteristics. Along this line, Queiroz et al. (2015) found greater magnitude of PREH in hypertensives than normotensives, and greater PREH has been reported after dynamic resistance exercises involving larger muscle mass (Polito and Farinatti, 2009) and multiple sets (Polito and Farinatti, 2009; De Brito et al., 2014; Figueiredo et al., 2015b). Nevertheless, the influence of these factors on PREH has been mainly defined based on comparisons of mean responses (i.e. comparing averages between conditions: hypertensives vs. normotensives; larger vs. smaller mass; and single vs. multiple sets) and not analysing whether there is a real interindividual variation in the response to exercise.

Recently, in the exercise physiology research field, a growing interest has showed up regarding the investigation of the interindividual responses to exercise, which may be relevant for individualizing exercise prescription (Hecksteden et al., 2015). For example, a former study (Lima et al., 2015) explored the interindividual variability of PREH and showed that 46 and 38% of the subjects actually presented systolic and diastolic PREH, respectively. However, this study provided no information about the heterogeneity of the individuals’ responses to a control intervention, which is relevant since it has been proposed (Atkinson and Batterham, 2015; Hopkins, 2015) that a true interindividual variation in physiological responses to exercise can only be accepted if the variation (i.e. standard deviation) of the changes in the exercise condition is larger than obtained in a control condition. Importantly, only when a true interindividual variation is demonstrated, it is logical to perform analyses regarding the individual responses, such as identifying responders to exercise or exploring predictors of responsiveness (Atkinson and Batterham, 2015; Hopkins, 2015). Nevertheless, to the best of our knowledge, no previous study has investigated the magnitude of interindividual variation of PREH before evaluating responsiveness or predictors.

Considering this background, it could be noted that despite PREH occurrence has already been well documented in the literature, its magnitude seems to vary among the subjects (i.e. interindividual variation), being important to detect whether this variation actually occurs using appropriate and new statistical approaches and, if so, which factors can influence this response. Therefore, the current study was designed to explore the interindividual variation of PREH and its predictors using a two-step approach. First, the magnitude of the interindividual variation in PREH was determined to confirm whether there was a variation among the subjects’ responses. Second, analyses were carried out to explore the potential predictors of PREH, considering factors related to the individuals’ and the exercise protocol’s characteristics.

## Materials and Methods

### Experimental Design

This is a retrospective study that pooled data from seven previous trials (Rezk et al., 2006; Queiroz et al., 2009, 2013, 2015; Teixeira et al., 2011; Prista et al., 2013; Freire et al., 2018) conducted by two different research laboratories (i.e. the Exercise Hemodynamic Laboratory from the School of Physical Education and Sport of the University of São Paulo and the Research Group on Acute and Chronic Effects of Exercise from the Department of Physical Education of the Federal University of Rio Grande do Norte) between 2006 and 2018.

The included trials attended the following criteria as: (1) were designed to evaluate PREH; (2) presented at least one dynamic resistance exercise session and one control session; (3) had clinic BP as outcome (i.e. BP measurements taken at rest before and after the exercise and control interventions); (4) employed a crossover design; (5) evaluated subjects free of cardiovascular disease except for arterial hypertension; and (6) evaluated subjects not receiving anti-hypertensive medications.

### Data Analysis

From each trial, the following individuals’ characteristics were extracted for each subject: gender (male or female); age (years); body mass index (BMI, kg/m^2^); diagnosis of hypertension (presence or absence); and baseline BP (defined as the average of the clinic BP values assessed before the exercise and control interventions). Additionally, the following exercise protocol data were extracted: time of day (morning or afternoon/evening); exercise intensity (≥70% of 1RM or<70% of 1RM; Casonatto et al., 2016); number of exercises (≥7 or<7; Queiroz et al., 2015); number of sets (single or multiple); and number of repetitions (≥12 or<12; i.e. median value of the current data). Resistance exercise volume was calculated by the product between the number of exercises, number of sets per exercise and number of repetitions per set, being classified as high ≥150 or low <150, which is a cut-off point previously employed in a PREH’s meta-analysis (Casonatto et al., 2016). Total exercise load was calculated by the product between exercise volume and exercise intensity, being classified as high ≥105 or low ≤105, which corresponds to the previous exercise volume of 150 multiplied by the previous intensity of 70% 1RM.

Finally, PREH was calculated for each subject in each trial by the net effect of the exercise, i.e. the difference between BP responses observed in the exercise and the control sessions, calculated as: PREH net effect=[(post-exercise BP – baseline BP in the exercise session) – (post-control BP – baseline BP in the control session)] (Fecchio et al., 2020).

To avoid duplicated data from the same subject, the following procedures were adopted. In trials that had evaluated post-exercise BP at multiple moments (e.g. 45, 60 and 90min after the exercise), the moment of greatest PREH was considered for data analysis. Regarding the trials that had compared different sessions of dynamic resistance exercise (e.g. exercises with different intensities), the session used for data analysis was raffled to avoid any selection bias.

### Statistical Analyses

Data distribution was confirmed by Shapiro-Wilk tests. PREH occurrence in the whole sample was checked by comparing the net effect with zero using paired t-tests.

The interindividual variation of PREH (aim 1) was calculated by the standard deviation of the individual responses (*SD*_IR_) as previously reported (Atkinson and Batterham, 2015; Hopkins, 2015). *SD*_IR_ represents the true magnitude of the interindividual variation of PREH adjusted for the random variations derived from biological and measurement sources, being calculated by the formula:$SD_{\mathrm{IR}}=\sqrt{\left(SD_{{\mathrm{ercercise}}^{2}}^{}-SD_{{\mathrm{control}}^{2}}^{}\right)}$ where *SD*_exercise_ and *SD*_control_ are the standard deviations of BP responses (i.e. the difference between BP measured before and after the intervention) observed in the exercise and the control sessions, respectively. Then, to a qualitative evaluation of the variation magnitude, *SD*_IR_ was expressed in standardised units, calculated by dividing *SD*_IR_ by the standard deviation of baseline BP and the results were interpreted using the following cut-off points: <0.30=low; 0.30 to 0.59=moderate; and>0.60=high variation (Hopkins, 2015).

When moderate or high variations were found, further analyses were conducted to explore the potential predictors of PREH (aim 2) using simple and multiple linear regressions. Firstly, attendance to statistical assumptions of linear regression modelling were checked. Linear relationship between continuous independent factors and PREH as well absence of heteroscedasticity were checked by scatter graphs. Normal distribution of standardised residuals was checked through histograms of residuals and normal probability plots. Independence of residuals was assessed by Durbin-Watson test accepting values between 1.0 and 3.0. Absence of multi-collinearity among the variables was confirmed by tolerance >0.1 and variance inflation factor<10.0. The presence of outliers was checked by the standardised predicted values and residuals, and a minimal sample size of 10 subjects for each independent factor was attended. Afterwards, single regression analyses were performed considering PREH (net effect) as the dependent variable and the individuals’ (gender, age, BMI, hypertension diagnosis and baseline BP) and the exercise protocol’s characteristics (intensity, number of exercises, number of sets, number of repetitions, exercise volume, total exercise load and time of day) as the independent variables. For the multiple regression analyses, a hierarchical modelling was performed with the independent variables clustered into two blocks: the primary block involved the individuals’ characteristics, and the secondary block included the exercise protocol’s characteristics. The inclusion of the variables in the multivariate model was performed with the forward method within each cluster.

Additionally, the proportion of responders and non-responders regarding the occurrence of PREH was calculated. For that, first, the typical error (TE) of BP measurement was calculated as: $\mathrm{TE}=SD_{\mathrm{difference}}/\sqrt{2}$, where *SD*_difference_ is the standard deviation of the differences in BP measured before the interventions in the exercise and control sessions (Hopkins, 2000). Then, subjects who presented PREH greater than TE were classified as responders (Swinton et al., 2018).

Statistical analyses were conducted using the Statistical Package for the Social Sciences for Windows (IBM SPSS Statistics, version 20) and the significance level was set as *p*≤0.05. Continuous data were reported as mean value ± standard deviation.

## Results

This study included 131 subjects, mainly non-elderly (95.4%), males (62.6%), nonobese (92.3%) and without hypertension (84.0%; Table 1). Regarding the dynamic resistance exercise protocols, most of the subjects executed protocols of low-intensity (82.4%), multiple sets (83.2%), high exercise volume (73.3%) and high total exercise load (70.2%), while almost half of them executed exercises in the evening (59.5%), with seven or more exercises (48.1%) and 12 or more repetitions per set (46.6%). All trials measured BP by the auscultatory method and with the subjects resting in the seated position. In five trials, post-exercise BP measurements were taken 60min after the exercise while in the other two trials, BP was measured at 45 and 90min. The occurrence of PREH in the whole sample (group analysis) was confirmed by significant net effects found for systolic (−6.8±8.1mmHg, *p*<0.001) and diastolic (−3.3±7.1mmHg, *p*<0.001) BPs.

**Table 1:** Descriptive data of the sample (*n*=131).

	Value
**Characteristics of individuals**
Male, n	82
Age, ys	36±15
Body mass index, kg/m^2^	24.4±3.4
Hypertension diagnosis, n	21
Baseline systolic BP, mmHg	114±12
Baseline diastolic BP, mmHg	73±11
**Characteristics of exercise protocol**
Exercise intensity	
< 70% of 1 RM, n	108
≥ 70% of 1 RM, n	23
Number of exercises	
< 7 exercises, n	68
≥ 7 exercises, n	63
Number of sets	
Simple sets, n	22
Multiple sets, n	109
Number of repetitions	
< 12, n	70
≥ 12, n	61
Exercise volume	
Low, n	35
High, n	96
Total exercise load	
Low, n	39
High, n	92
Time of day	
Morning, n	53
Evening, n	78

*Continuous values are expressed as mean±standard deviation. BP, blood pressure; RM, repetition maximum. Exercise volume was calculated by the product between number of exercises, number of sets per exercise and number of repetitions per set, being denoted as high when≥150. Total exercise load was calculated by the product between exercise volume and intensity, being denoted as high when≥105*.

The results related to the quantification of the interindividual variation of PREH (aim 1) are shown in Table 2. *SD*_IR_ for SBP was 4.4mmHg and 0.35 standardised units, revealing a moderate variation. For DBP, *SD*_IR_ was 3.6mmHg and 0.32 standardised units, also revealing a moderate variation.

**Table 2:** Quantification of the interindividual variation of post-dynamic resistance exercise hypotension.

	BP response to exercise session	BP response to control session	*SD*_IR_ mmHg (95%CI)	*SD*_IR_ standardised units (95%CI)	Classification
SBP	−5.3±7.3	1.5±5.9	4.4 (1.9 to 5.9)	0.35 (0.15 to 0.47)	Moderate
DBP	−0.1±5.6	3.1±4.3	3.6 (2.0 to 4.7)	0.32 (0.18 to 0.42)	Moderate

*Values are mean±SD; BP, blood pressure; CI, confidence level; SD_IR_, standard deviation of the individual responses; SBP, systolic blood pressure; and DBP, diastolic blood pressure*.

Regarding aim 2, simple linear regressions (Supplementary Figure 1) showed a significant association of systolic PREH only with baseline SBP (*B*=−0.131, *p*=0.025). In the multivariate analysis (Table 3), baseline SBP and number of sets were included in the final model as: systolic PREH=14.374–0.157 (baseline SBP) – 3.910 (multiple sets: yes=1, no=0); *R*^2^=0.069; *p*=0.010. For diastolic PREH (Supplementary Figure 2), simple linear regressions showed significant associations with baseline DBP (*B*=−0.215; *p*<0.001), hypertension diagnosis (*B*=−3.797; *p*=0.024), male gender (*B*=−3.862; *p*=0.002), high exercise volume (*B*=−4.844, *p*<0.001) and high total exercise load (*B*=−4.162, *p*=0.002). In the multivariate analysis (Table 4), baseline DBP and high exercise volume were included in the final model as: Diastolic PREH=13.680–0.191 (baseline DBP) – 4.212 (high exercise volume: yes=1, no=0); *R*^2^=0.174; *p*<0.001.

**Table 3:** Multiple linear regression assessing predictors of post-dynamic resistance exercise hypotension for systolic blood pressure (SBP).

	Coefficient B (unstandardized)	Coefficient β (standardised)	Value of *P*
**Multivariate model (*R***^**2**^ **=0.069)**	–	–	0.010^*^
Intercept	14.374±7.221	–	0.049^*^
Baseline SBP (mmHg)	−0.157±0.059	−0.233	0.008^*^
Multiple sets (yes or no)	−3.910±1.891	−0.180	0.041^*^
**Variables Excluded from the Model**			
Male gender (yes or no)	–	−0.057	0.592
Age (years)	–	−0.016	0.858
BMI (kg/m^2^)	–	−0.007	0.939
Hypertension diagnosis (yes or no)	–	0.053	0.610
Exercise intensity ≥70%1RM (yes or no)	–	0.077	0.380
N. exercises ≥7 exercises (yes or no)	–	−0.084	0.429
N. repetitions ≥12 (yes or no)	–	0.011	0.910
High exercise volume (yes or no)		0.060	0.662
High total exercise load (yes or no)		0.041	0.738
Evening (yes or no)	–	0.051	0.587

*BMI, body mass index; RM, repetition maximum; N, number; High exercise volume≥150; High total exercise load≥105*;

* *significant (p<0.05)*.

**Table 4:** Multiple linear regression assessing predictors of post-dynamic resistance exercise hypotension for diastolic blood pressure (DBP).

	Coefficient B (unstandardized)	Coefficient β (standardised)	Value of *P*
**Multivariate Model (*R***^**2**^ **=0.174)**			<0.001^*^
Intercept	13.680±3.912	–	0.001^*^
Baseline DBP (mmHg)	−0.191±0.053	−0.289	0.001^*^
High exercise volume (yes or no)	−4.212±1.295	−0.264	0.001^*^
**Variables Excluded from the Model**			
Male gender (yes or no)	–	−0.162	0.081
Age (years)	–	0.107	0.216
BMI (kg/m^2^)	–	0.013	0.885
Hypertension diagnosis (yes or no)	–	−0.132	0.249
Exercise intensity ≥70%1RM (yes or no)	–	−0.054	0.551
N. of exercises ≥7 exercises (yes or no)	–	0.160	0.090
Multiple sets (yes or no)		0.005	0.969
N. repetitions ≥12 (yes or no)	–	0.005	0.947
High total exercise load (yes or no)	–	0.047	0.828
Evening (yes or no)	–	−0.008	0.922

*BMI, body mass index; RM, repetition maximum; N, number; High exercise volume≥150; High total exercise load≥105*;

* *significant (p<0.05)*.

**Figure 1:**
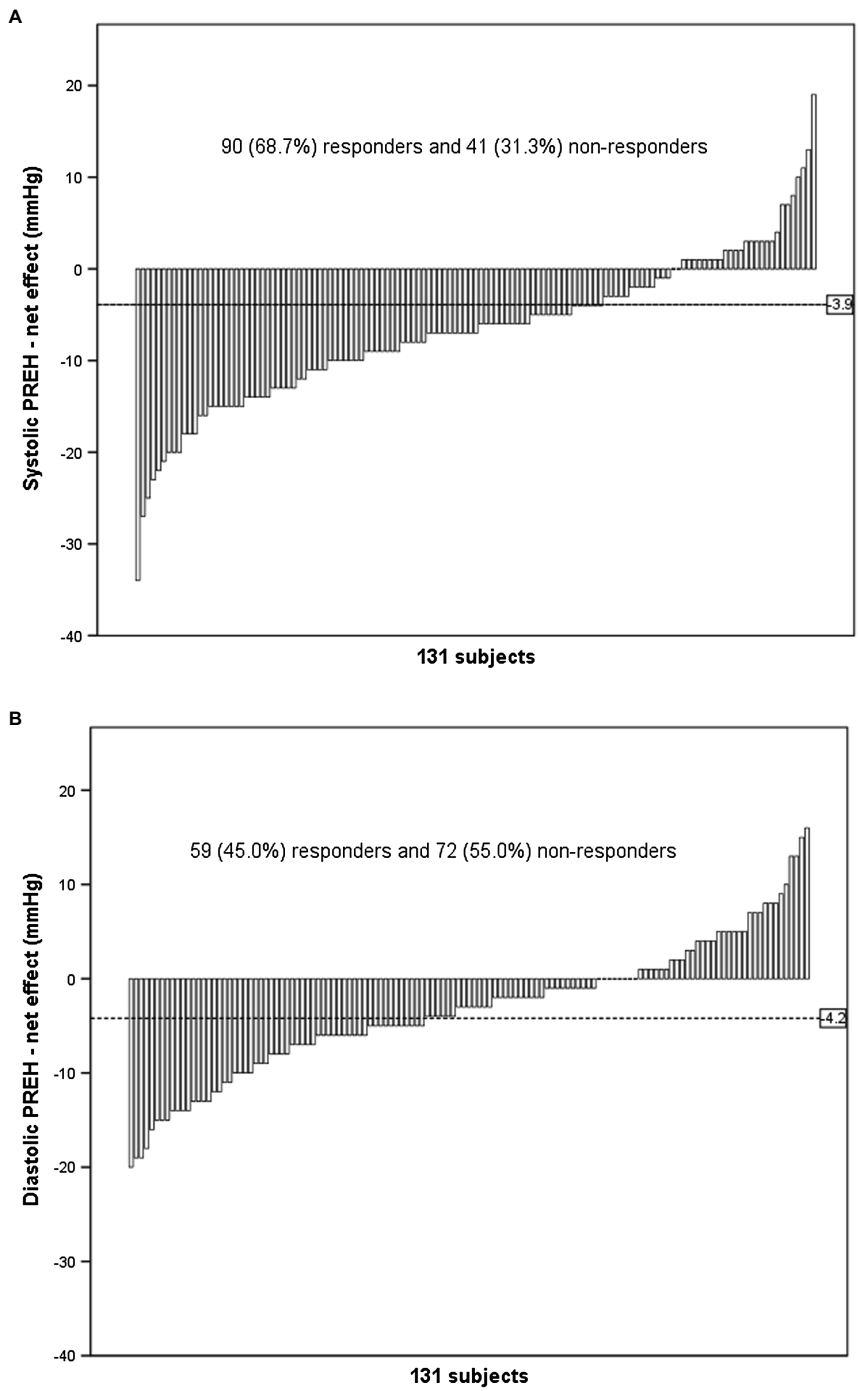
Individual responses of post-dynamic resistance exercise (PREH) calculated by the exercise net effect [(post-exercise BP – baseline BP in the exercise session) – (post-control BP – baseline BP in the control session)]. The dashed line represents the typical error of measurement and individuals that presented PREH greater than this threshold were classified as responders.

Lastly, for the analyses of responders, TEs of baseline SBP and DBP were, respectively, 3.9 and 4.2mmHg. Thus, 41 subjects (31.3%) were classified as non-responders regarding systolic PREH, whereas 72 subjects (55.0%) were classified as non-responders for diastolic PREH (Figure 1).

## Discussion

The current study has two main findings. First, there is a moderate interindividual variation in PREH magnitude as demonstrated by *SD*_IR_’s results. Second, systolic PREH is mainly influenced by baseline BP and the number of sets executed during the dynamic resistance exercise session, while diastolic PREH is mainly influenced by baseline DBP and the exercise volume performed as demonstrated by multiple linear regression analyses.

As consistently reported in the current literature (Casonatto et al., 2016), the occurrence of PREH was also observed in the whole sample of the present study by the significant net effects demonstrated for SBP (*p*<0.001) and DBP (*p*<0.001). The novelty of the current study was to perform a robust quantification of the interindividual variation of PREH using recommended statistical approaches (Atkinson and Batterham, 2015; Hopkins, 2015). In this sense, the present analyses confirmed the existence of a moderate interindividual variation in PREH for both SBP and DBP based on *SD*_IR_ expressed in standardised units being between 0.30 and 0.59. Indeed, the current results show a considerable variation in PREH magnitude across the subjects for both systolic (net effect ± *SD*_IR_=−11.2 to −2.4mmHg) and diastolic PREH (net effect ± *SD*_IR_=−6.9 to 0.3mmHg). In addition, this pioneer demonstration of real interindividual variation on PREH allowed the exploration of its predictors related to individuals’ and exercise protocol’s characteristics.

Along this line, the current study firstly performed simple regression analyses to investigate individuals’ characteristics associated with PREH, which showed greater systolic PREH associated with higher baseline SBP, whereas greater diastolic PREH was associated with higher baseline DBP, hypertension diagnosis and male gender. However, hypertension diagnosis and male gender were not maintained in the multivariate model, suggesting that their associations with PREH might be dependent of another factor or factors. In fact, baseline BP was the only investigated individual’s characteristic that predicted PREH magnitude in the multivariate analysis, with higher baseline BP being associated with greater PREH. Previous original studies (Melo et al., 2006; Queiroz et al., 2009) have also reported significant associations between BP decrease after a dynamic resistance exercise session and the pre-exercise or baseline BP. Thus, the current result strengthens this finding by analysing data from a larger sample and employing specific statistical approaches. Importantly, the existence of such association may have clinical relevance, revealing a greater effectiveness of dynamic resistance exercise in individuals with altered BP who may benefit more from this acute post-exercise BP-lowering effect (Kenney and Seals, 1993).

Regarding the influence of exercise protocol’s variables, the number of sets and the exercise volume were significant predictors of PREH, suggesting that a greater amount of exercise is associated with a greater PREH. This result contrasts with previous meta-analytic data (Casonatto et al., 2016) that found no influence of exercise volume (i.e. exercises ^*^sets ^*^repetitions) on PREH. The discrepancy may reflect the higher sensitivity of the statistical analyses performed with individual participant data, as used in the present study, to detect predictors’ effects (Tierney et al., 2015). The greater hypotensive effect induced by protocols with higher volume may be related to a greater effect on vasculature since De Brito et al. (2014) showed a bigger decrease in forearm vascular resistance after a session of dynamic resistance exercise with higher volume (i.e. multiple versus single sets), which also resulted in a greater PREH. Differently from variables related to exercise volume, the current results did not reveal exercise intensity as a predictor of PREH. Indeed, the role of exercise intensity on PREH is controversial in the literature with original studies reporting greater PREH after high- (Duncan et al., 2014; De Brito et al., 2015a), moderate- (Figueiredo et al., 2015a) and low- (Rezk et al., 2006) intensity exercises as well as no difference between different intensities (Cavalcante et al., 2015). Lastly, the current study did not find a significant association between the time of day in which exercise was executed and PREH although such influence has been reported for aerobic exercise (De Brito et al., 2015b). Nevertheless, to the better of our knowledge, no previous study has directly compared PREH after morning and evening exercise.

The present results might have important clinical implications. Besides confirming an interindividual variability on PREH’s responses, the study showed that an important fraction of the subjects did not present a relevant decrease in BP after resistance exercise (i.e. 31.3% were not responders for SBP and 55.5% for DBP), highlighting the importance of optimising exercise protocol for inducing PREH, which can be done based on the current results. In this sense, the multivariate regression analyses revealed a greater importance of exercise volume than intensity to optimise PREH, since exercise volume or sets but not total exercise load or intensity were significant predictors. In fact, the final multivariate regression models showed that exercise protocols composed by multiple sets and high exercise volume (at least 150) can potentiate systolic and diastolic PREH in approximately 4mmHg. Importantly, it has been proposed that regular exposure to greater PREH episodes might optimise chronic BP reductions after training (Luttrell and Halliwill, 2015; Brito et al., 2018). Therefore, to increase PREH magnitude, the present study results suggest the employment of training protocols with more exercises, more sets, and more repetitions per set.

Besides employing a robust interindividual statistical technique, the main strength of the present study was to have pooled data from seven trials, including a sample of 131 individuals with different characteristics and that executed different protocols of dynamic resistance exercises, allowing for a comprehensive exploration of PREH occurrence, variation, and predictors. On the other hand, it is important to mention some limitations. First, the study is limited by its retrospective design that confines the analyse only to the predictors included in the original trials and the characteristics addressed in each one of them. However, the analysis of interindividual variation and predictors requires a substantial sample size (Hopkins, 2015) that is difficult to achieve in single prospective trials. Second, the present study opted to analyse the greatest BP decrease after the exercise, limiting the results to the interindividual variation of the greatest PREH. The inclusion of BP measurements performed at different time points after the exercise can be suggested as a bias. However, in a complementary analysis considering data collected at 60min after the interventions (*n*=106, data not shown), PREH variation remained moderate (SBP *SD*_IR_=0.34 and DBP *SD*_IR_=0.32 standardised units). Third, the multivariate models presented *R*^2^ of 0.069 and 0.174, respectively, for SBP and DBP, explaining 7 and 17% of the PREH variations, which suggests that factors beyond those covered in the original studies included in this analysis, such as hydration status, genetic polymorphisms, race, muscle mass involved in exercise or others, may also affect PREH magnitude and should be investigated by future research. Finally, some caution is needed regarding the extrapolation of the current findings to other situations. In this sense, the present results cannot be transferred to other clinical populations, such as patients with cardiovascular disease, because they may present different cardiovascular dysfunctions that may impose a greater variation in PREH with different predictors. Additionally, results cannot be extrapolated for other types of exercise, such as aerobic exercise, in which an increase in volume prolongs a constant cardiovascular load instead of promoting a progressive cardiovascular load as observed in dynamic resistance exercise, this difference may induce a different impact on PREH variation. Future studies should investigate the interindividual variation of BP responses in these situations.

## Conclusion

PREH presents a considerable interindividual variation with its magnitude being influenced by baseline BP, number of exercise sets and exercise volume.

## Data Availability Statement

The original contributions presented in the study are included in the article/**Supplementary Material**, and further inquiries can be directed to the corresponding author.

## Ethics Statement

Ethical review and approval were not required for retrospective study on human participants in accordance with the local legislation and institutional requirements as original studies included in analysis have already been approved by ethics committees. Written informed consent for participation was obtained for the oiriginal trials included in this study.

## Author Contributions

RF: conception, design, statistical analysis, interpretation of data, and writing of the manuscript. AQ: extraction of data and revision of the manuscript. RR-D and EC: conception, design, extraction, interpretation of data, and revision of the manuscript. CF: conception, design, interpretation of data, revision of the manuscript, and final approval of the version to be submitted. All authors contributed to the article and approved the submitted version.

## Funding

This work was supported by the Brazilian National Council for Scientific and Technological Development (CNPQ, process 304436/2018-6), the São Paulo State Research Foundation (FAPESP, process 2018/12390-1 and process 2018/23653-3) and the Coordination for the Improvement of Higher Education – Brazil (CAPES, 0001).

## Conflict of Interest

The authors declare that the research was conducted in the absence of any commercial or financial relationships that could be construed as a potential conflict of interest.

## Publisher’s Note

All claims expressed in this article are solely those of the authors and do not necessarily represent those of their affiliated organizations, or those of the publisher, the editors and the reviewers. Any product that may be evaluated in this article, or claim that may be made by its manufacturer, is not guaranteed or endorsed by the publisher.

## Supplementary Material

The Supplementary Material for this article can be found online at: https://www.frontiersin.org/articles/10.3389/fphys.2021. 787444/full#supplementary-material

Supplementary Figure 1 Simple linear regression analyses assessing possible predictors of systolic post-dynamic resistance exercise hypotension (PREH) related to the following characteristics of the individuals and exercise protocol: baseline systolic blood pressure, SBP **(A)**; gender **(B)**; age **(C)**; body mass index, BMI **(D)**; hypertension diagnosis **(E)**; daytime of exercise **(F)**; exercise intensity **(G)**; number of exercises **(H)**; number of sets **(I)**; number of repetitions **(J)**; exercise volume **(K)**; and total exercise load **(L)**. RM, repetition maximum. ^*^Significant moderator (*p*<0.05). Dashed line represents the typical error of SBP (−3.9mmHg) and individuals below this line are responders.

Supplementary Figure 2 Simple linear regression analyses assessing possible predictors of diastolic post-dynamic resistance exercise hypotension (PREH) related to the following characteristics of the individuals and exercise protocol: baseline diastolic blood pressure, DBP **(A)**; gender **(B)**; age **(C)**; body mass index, BMI **(D)**; hypertension diagnosis **(E)**; daytime of exercise **(F)**; exercise intensity **(G)**; number of exercises **(H)**; number of sets **(I)**; number of repetitions **(J)**; exercise volume **(K)**; and total exercise load **(L)**. RM, repetition maximum. ^*^Significant moderator (*p*<0.05). Dashed line represents the typical error of DBP (−4.2mmHg) and individuals below this line are responders.
