# Supplementary figures and images for: Post-dynamic Resistance Exercise Hypotension: Exploring Individual Responses and Predictors
Source: Front Physiol. 2021 Dec 2;12:787444. doi: 10.3389/fphys.2021.787444 (PMC9126191; doi:10.3389/fphys.2021.787444)

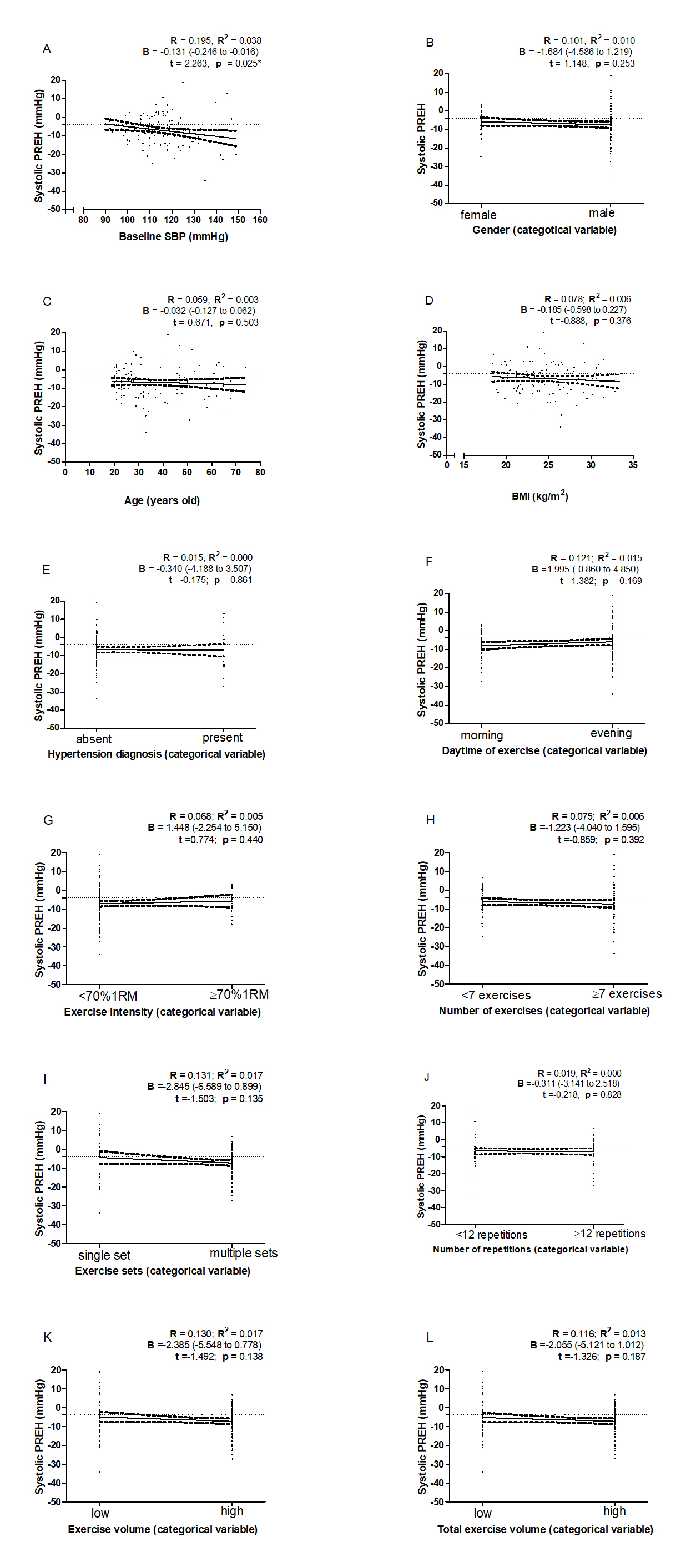

Supplement: Supplementary Figure 1 — Simple linear regression analyses assessing possible predictors of systolic post-dynamic resistance exercise hypotension (PREH) related to the following characteristics of the individuals and exercise protocol: baseline systolic blood pressure, SBP (A); gender (B); age (C); body mass index, BMI (D); hypertension diagnosis (E); daytime of exercise (F); exercise intensity (G); number of exercises (H); number of sets (I); number of repetitions (J); exercise volume (K); and total exercise load (L). RM, repetition maximum. *Significant moderator (p<0.05). Dashed line represents the typical error of SBP (−3.9mmHg) and individuals below this line are responders. [file image_1.tiff]

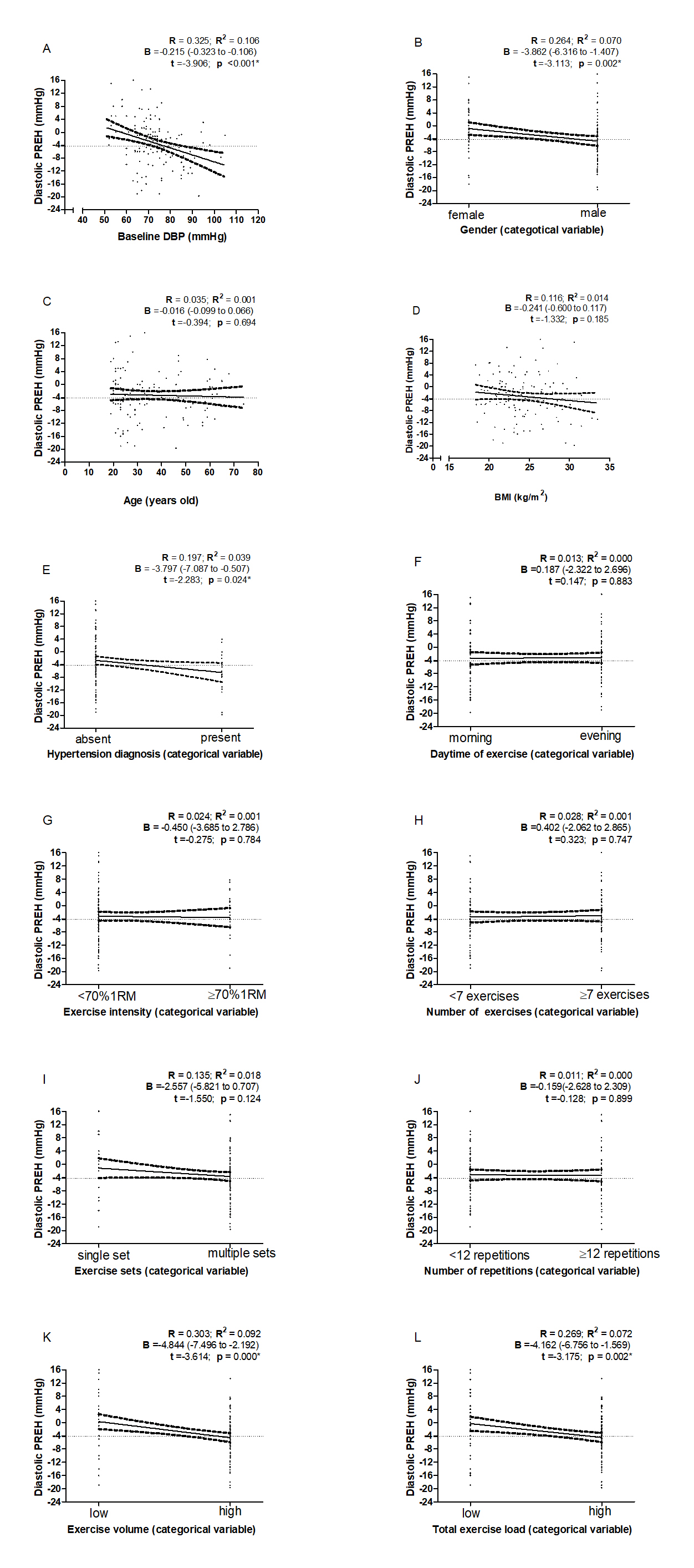

Supplement: Supplementary Figure 2 — Simple linear regression analyses assessing possible predictors of diastolic post-dynamic resistance exercise hypotension (PREH) related to the following characteristics of the individuals and exercise protocol: baseline diastolic blood pressure, DBP (A); gender (B); age (C); body mass index, BMI (D); hypertension diagnosis (E); daytime of exercise (F); exercise intensity (G); number of exercises (H); number of sets (I); number of repetitions (J); exercise volume (K); and total exercise load (L). RM, repetition maximum. *Significant moderator (p<0.05). Dashed line represents the typical error of DBP (−4.2mmHg) and individuals below this line are responders. [file image_2.tiff]
